# Supplementary material for: Empirical methods for controlling false positives and estimating confidence in ChIP-Seq peaks
Source: BMC Bioinformatics. 2008 Dec 5;9:523. doi: 10.1186/1471-2105-9-523 (PMC2628906; doi:10.1186/1471-2105-9-523)
Supplement: Additional File 1 — A variety of html documents from the USeq web site detailing the available applications, their best usage, output file type descriptions, command line menus, etc. [file 1471-2105-9-523-S1.zip › USeqUserGuides/JavaDoc.zip!/JavaDoc/bioroot/class-use/LabGroup.html]

Uses of Class bioroot.LabGroup


|  |  |  |
| --- | --- | --- |
| **Overview** \| **Package** \| **Class** \| **Use** \| **Tree** \| **Deprecated** \| **Index** \| **Help** | |  |
| PREV   NEXT | **FRAMES**    **NO FRAMES**     **All Classes** |  |


---


## **Uses of Class bioroot.LabGroup**

| Packages that use LabGroup | |
| --- | --- |
| **bioroot** |  |

Uses of LabGroup in bioroot

| Methods in bioroot that return LabGroup | |
| --- | --- |
| `static LabGroup[]` | `CronArchive.getLabGroupsToArchive(DBUtil bioRoot)`             Identifies lab groups that should be archived based on their last archive date and schedule. |

| Methods in bioroot with parameters of type LabGroup | |
| --- | --- |
| `static java.lang.String` | `CronArchive.getMessage(LabGroup labGroup, boolean noRecords)` |
| `static void` | `CronArchive.loadSuperUserEmailAddresses(DBUtil bioRoot, LabGroup[] labGroups)`             Loads an array of LabGroup[] with superuser email addresses. |
| `static boolean` | `CronArchive.setLastArchiveDate(LabGroup[] labGroups, DBUtil bioRoot)`             If archived will set that labgroups lastArchive to today |

---


|  |  |  |
| --- | --- | --- |
| **Overview** \| **Package** \| **Class** \| **Use** \| **Tree** \| **Deprecated** \| **Index** \| **Help** | |  |
| PREV   NEXT | **FRAMES**    **NO FRAMES**     **All Classes** |  |


---
